# An Efficient Triplex TaqMan Quantitative PCR to Detect a Blackleg-Causing Lineage of *Pectobacterium brasiliense* in Potato Based on a Pangenome Analysis

**DOI:** 10.3390/microorganisms11082080

**Published:** 2023-08-13

**Authors:** Theo A. J. van der Lee, Marga P. E. van Gent-Pelzer, Eef M. Jonkheer, Balázs Brankovics, Ilse M. Houwers, Jan M. van der Wolf, Peter J. M. Bonants, Inge van Duivenbode, Robert A. M. Vreeburg, Mathijs Nas, Sandra Smit

**Affiliations:** 1 Biointeractions and Plant Health, Wageningen Plant Research, Droevendaalsesteeg 1, 6708 PB Wageningen, The Netherlands; 2 Bioinformatics Group, Wageningen University, Droevendaalsesteeg 1, 6708 PB Wageningen, The Netherlands; 3 Dutch General Inspection Service (NAK), Randweg 14, 8304 AS Emmeloord, The Netherlands

**Keywords:** *Pectobacterium brasiliense*, TaqMan, field testing, pangenome, blackleg

## Abstract

*P. brasiliense* is an important bacterial pathogen causing blackleg (BL) in potatoes. Nevertheless, *P. brasiliense* is often detected in seed lots that do not develop any of the typical blackleg symptoms in the potato crop when planted. Field bioassays identified that *P. brasiliense* strains can be categorized into two distinct classes, some able to cause blackleg symptoms and some unable to do it. A comparative pangenomic approach was performed on 116 *P. brasiliense* strains, of which 15 were characterized as BL-causing strains and 25 as non-causative. In a genetically homogeneous clade comprising all BL-causing *P. brasiliense* strains, two genes only present in the BL-causing strains were identified, one encoding a predicted lysozyme inhibitor Lprl (LZI) and one encoding a putative Toll/interleukin-1 receptor (TIR) domain-containing protein. TaqMan assays for the specific detection of BL-causing *P. brasiliense* were developed and integrated with the previously developed generic *P. brasiliense* assay into a triplex TaqMan assay. This simultaneous detection makes the scoring more efficient as only a single tube is needed, and it is more robust as BL-causing strains of *P. brasiliense* should be positive for all three assays. Individual *P. brasiliense* strains were found to be either positive for all three assays or only for the *P. brasiliense* assay. In potato samples, the mixed presence of BL-causing and not BL-causing *P. brasiliense* strains was observed as shown by the difference in Ct value of the TaqMan assays. However, upon extension of the number of strains, it became clear that in recent years additional BL-causing lineages of *P. brasiliense* were detected for which additional assays must be developed.

## 1. Introduction

Bacteria from the *Pectobacterium* genus are in the top ten of scientifically or economically most relevant plant pathogenic bacteria [1]. They are causal to a spectrum of bacterial soft rot diseases including soft rot, blackleg, and stem wilt, in a wide host range of important crops [2,3,4]. Among the 19 *Pectobacterium* species described [4,5], *P. brasiliense* has considerably increased in importance as a pathogen of various crops in different geographic locations. *P. brasiliense* was initially described as an atypical *Erwinia carotovora* strain causing blackleg on potato tubers in Brazil [6]. Subsequently, it was described as a subspecies of *P. carotovorum*, until recently when it was elevated to the species level on the basis of whole genome sequence analysis [7]. The bacterium causes significant losses in important crops such as potato, tomato, beet, cabbage, pepper, and tobacco [4]. In potato, *P. brasiliense* has been found on all continents. In 2014, the pathogen was recorded for the first time in Europe [8]. Since 2016, it has become the most prevalent blackleg-causing pathogen in the Netherlands [9]. This increasing prevalence and damage caused by *P. brasiliense* prompted the need for efficient detection tools that can be used in management strategies, in particular in the testing of propagation material, and in research. For this reason, a conventional PCR and two TaqMan assays were developed for the detection of *P. brasiliense* [6,10,11]. Recent results indicated that plants grown from seed lots infected with *P. brasiliense* frequently remained symptomless, indicating the existence of strains that do not cause blackleg symptoms (Vreeburg, NAK, Emmeloord, the Netherlands, personal communication). Subsequent field tests with a range of strains demonstrated that most *P. brasiliense* strains could be separated into two groups, one of them genetically homogeneous and containing strains showing a high ability to cause BL and a second, more diverse group of strains that were unable to cause BL [12]. Consequently, an assay that would specifically detect strains that have the ability to cause blackleg of *P. brasiliense* would be of added value.

In this study, a comparative genomic approach was used to identify candidate regions that could also be functionally related to the ability to cause blackleg. Advances in next-generation sequencing technologies (NGS) have made the reconstruction of genomes easier and more accessible over the years. The diversity that was found among strains of the same species resulted in the concept of a pangenome, which reflects a bacterial species more accurately than any single member can. The pangenome is an abstract representation of the genomes of all the strains that are present in the population, species, or genus. Such inclusive pangenomes provide novel insights into the organization and evolution of dynamic genomes. The pangenomic data analysis platform, PanTools [13], has a hierarchical data structure, including sequence data (represented as a localized, compressed De Bruijn graph), structural/functional annotations, and crosslinks between DNA and protein sequences and annotations. Recently this platform was extended with modules to study phylogeny and to incorporate and use phenotypic data [12,14]. In PanTools, a set of 15 BL-causing strains and 25 non-causative strains was analyzed, and 88 genes were identified that were specific for a blackleg-causing *P. brasiliense* clade, containing all BL-causing strains of *P. brasiliense* [12]. Here the further selection and application of the selected regions of those genes for diagnostics and detection are described. Based on a putative function, two genes were selected that were only present in the BL-causing *P. brasiliense* clade. A gene encoding a predicted lysozyme inhibitor Lprl (LZI) and one encoding a putative Toll/interleukin-1 receptor (TIR) domain-containing protein. Specific TaqMan assays for BL-causing isolates were designed and subsequently adapted to fit into a triplex TaqMan assay that allows simultaneous detection. The assay was tested on strains with a known BL-causing phenotype and applied in symptomatic potato tissue and screenings of asymptomatic tubers for the detection of BL-causing *P. brasiliense*. Testing of additional field strains revealed more diversity than originally included in the pangenome analysis, and the implications for the detection using the designed TaqMan triplex are discussed.

## 2. Materials and Methods

### 2.1. Collection of Strains

Bacteria from the IPO collection, stored at −80 °C on beads (Protect bacterial preservers; Technical Service Consultants Ltd, Heywood, Lancashire, UK) were grown on tryptone soy agar (TSA; Oxoid, Hampshire, UK) for 48–72 h at 27 °C. *Pectobacterium* strains from the NAK collection, stored at −80 °C in 15% glycerol with half-strength nutrient broth, were grown on Nutrient Agar plates for 1 day at 28 °C. Strains are listed in Table S1. Non-symptomatic material was ground or crushed and incubated in pectate buffer [10] before spreading on single-layer Crystal Violet Pectate (CVP) plates [15]. Characteristic cavity-forming colonies were sub-cultured on CVP and nutrient agar plates to obtain pure strains.

### 2.2. Phenotyping Pectobacterium Strains

The ability of *Pectobacterium* strains to cause BL was assessed in seed potatoes and mini-tubers of the variety Kondor according to van der Wolf et al. [10]. Suspension of OD_600_ = 0.1 was prepared in 10 mM phosphate buffer pH 7.2 and diluted an extra 100×, resulting in a suspension of about 10^6^ cfu ml^−1^. Potato tubers were submerged in the solution and brought under −0.07 Pa vacuum. The vacuum was kept for 10 min, after which tubers were left submerged for another 15 min. Tubers were left to air-dry before planting. The ability to cause BL was scored as the development of typical plant symptoms as described previously [10].

### 2.3. Genome Sequencing

A total of 82 genomes were sequenced for our previous work [12] and this study (the ones with a Biosample accession in Table S2). For this study, additional available genome information, including that from replicated cultures and different sequencing strategies, was used for validation. From the sequenced genomes, one representative per strain was submitted to GenBank (NCBI accession sequenced genomes in Table S2). The other 35 genomes from the set of 117 were downloaded from GenBank.

DNA was extracted from pure bacterial cultures obtained by growing the strains on TSA medium. The DNA extraction for Illumina sequencing was performed by using the Wizard^®^ Magnetic DNA Purification System for Food (Promega, Leiden, The Netherlands) according to the manufacturer’s protocol. DNA for PacBio sequencing was obtained with the Gentra Puregene Yeast/Bacteria Kit (Qiagen, Hilden, Germany) following the manufacturer’s protocol. Quantification was carried out by measuring the samples with the Qubit Fluorometer using the Qubit dsDNA HS Assay Kit (ThermoFisher Scientific, Waltham, MA, USA). DNA samples were sequenced with the NovaSeq or Hiseq (Illumina, San Diego, CA, USA) or the PacBio Sequel (Pacific BioSciences, Menlo Park, CA, USA). The sequencing procedure was operated at the services of Business Unit Bioscience, Wageningen Plant Research (Wageningen, The Netherlands). Prior to sequencing, library preparations were performed by using the TruSeq Nano DNA Library Prep Kit (Illumina, San Diego, CA, USA). Sequencing resulted in 125-bp paired-end reads. The PacBio sequencing was performed using the SMRTbell Template Prep Kit 1.0 (Pacific BioSciences, Menlo Park, CA, USA).

### 2.4. Pangenome Construction, Annotation, and Analysis

A pangenome was constructed from 117 high-quality genomes (Table S2), 116 *P. brasiliense* and one *P. aquaticum*, with PanTools (v3.0.0, Bioinformatics Group, Wageningen University & Research, Wageningen, The Netherlands, https://git.wur.nl/bioinformatics/pantools, accessed on 11 August 2022) ‘build_pangenome’. The *P. aquaticum* type strain (A212-S19-A16) was included in the pangenome as an outgroup. All subsequent steps were performed with PanTools functionalities. Phenotypic data, such as the ability of the strain to cause BL, were linked to each genome via `add_phenotypes`. Gene models from which protein sequences were derived were included in the pangenome database using ‘add_annotations’. Proteins were clustered into homology groups using the ‘optimal_grouping’ function, which determines the optimal clustering parameters by assessing the grouping of BUSCO genes [16]. The first step in ‘core_phylogeny’ aligned sequences from single-copy orthologous groups in two consecutive rounds with MAFFT (v7.453, https://mafft.cbrc.jp/alignment/software/, accessed on 11 August 2022)). An initial alignment of protein sequences found the longest start and end gaps and trimmed the nucleotide input sequences accordingly. The second alignment was performed on the trimmed nucleotide sequences. Parsimony-informative SNPs (265,343) were identified from the 2371 trimmed alignments and concatenated into a single sequence per genome. The maximum likelihood (ML) phylogeny was inferred on the concatenated SNP sequences using IQ-tree (v1.6.12, http://www.iqtree.org/release/v1.6.12, accessed on 11 August 2022) [17] with default settings and 10,000 bootstrap iterations.

Various functional annotation databases were included in the pangenome graph using ‘add_functions’: Gene Ontology (GO) version June 2019 (http://geneontology.org, accessed on 11 August 2022), InterPro v74 (https://ebi.ac.uk/interpro, accessed on 11 August 2023), the Pfam v32 protein family database (http://pfam.xfam.org, accessed on 11 August 2022), and TIGRFAM release 15 (https://jcvi.org/tigrfams, accessed on 11 August 2022). This method used InterProScan output [18] to link gene nodes to their corresponding functional annotation. Furthermore, Phobius [19] signal peptides and transmembrane domain predictions were integrated into the pangenome graph.

The ‘gene_classification’ method was used to find genes significantly correlated with the BL-causing phenotype (Table S3), showing either presence or absence in the BL-causing or non-causative strains. The identified genes were manually checked in all genomes of the pangenome to confirm the correctness of the gene models. In addition, a local BLAST nucleotide database was created and searched to verify if genes were missing in the genome annotations. Subsequently, genes encoding for secreted proteins with a putative role in the BL-causing phenotype suggested by their functional annotation were further analyzed. Genes encoding for secreted proteins and with a possible role in the BL-causing phenotype were examined in more detail by BLAST searches and manual analysis, which resulted in the selection of two genes (Table S3: GJPMHAJK_01842 and GJPMHAJK_00450) for TaqMan assay design.

### 2.5. TaqMan Assay Design

Two sets of primers and probes for the TaqMan PCRs for the detection of BL-causing *P. brasiliense* strains were designed based on the selected two genes (LZI and TIR). Primers and probes were designed using the PrimerQuest^®^ Tool by IDT (Coralville, IA, USA). For the design, amplicons were tested for specificity using BLASTN, at which time no significant homology was found in the NCBI database. Primer and probe sequences and the sequence of the synthetic DNA target (gBlocks), including the previously published PcB primers for the detection of *P. brasiliense* [11], are listed in Table 1.

### 2.6. DNA Extraction for TaqMan Assay

DNA was isolated from bacterial strains listed in Table S1. DNA was extracted from pure cultures of bacteria grown for 48 h on TSA at 27 °C. The bacteria were scraped from the agar surface and stored at −20 °C until DNA extraction. The DNA was extracted using the Wizard Magnetic DNA Purification System for Food (Promega, Leiden, The Netherlands). DNA yield was determined by fluorescence using the Pico^®^ Green I dye (Invitrogen) and a TECAN Infinite^®^ M200 Pro microplate reader (Tecan Group Ltd., Männedorf, Switzerland).

For non-symptomatic samples, a random sample of 200 tubers was tested as four composite samples of 50 tubers at the Dutch General Inspection Service for Agricultural Seed and Seed Potatoes (NAK). The heel-end of each potato tuber was carefully removed using a sterile scalpel blade and macerated, and the extracts were enriched in Pectate Enrichment Broth for 72 h at 20 °C. After this bacterial enrichment, DNA was extracted using the Agowa sbeadex maxi plant kit (LGC, Teddington, UK) in conjunction with a KingFisher Flex Magnetic Particle Processor (Thermo Fisher Scientific, Carlsbad, CA, USA) according to the manufacturer’s instructions.

### 2.7. TaqMan Assay

TaqMan assays were performed at two different locations, Wageningen Plant Research (WPR) and Dutch General Inspection Service (NAK), using slightly different reaction conditions. The selected primers and probe sets with corresponding gBlocks were tested in TaqMan assays performed in a 96-well format in an ABI Quantstudio 12K flex apparatus (WPR) or the ABI 7500 (NAK). For each TaqMan assay, 1 μL sample was mixed with 24 μL reaction mix (WPR) or a 2 μL sample was mixed with 8 μL (NAK) resulting in a final concentration of 1× PerfeCTa qPCR ToughMix Low ROX (Quantabio, Beverly, MA, USA), 100 nM probe, and 300 nM of each forward and reverse primer. The reaction conditions were as follows: an initial incubation step at 95 °C for 2 min; 40 cycles of 95 °C for 15 s followed by 60 °C for 60 s. Analysis of the data was undertaken by automatic threshold calculation within the Quantstudio system software version 1.3 (WPR) or using a fixed threshold within the ABI 1.5 software version (NAK). Ct values were interpreted qualitatively applying a threshold of Ct < 30 or quantitatively using a standard curve with a ^10^log dilution series 10^6^ to 1 copy. In every run, water was used as negative control. The specificity of the TaqMan assays was evaluated using 0.4 ng DNA of pure strains.

## 3. Results

### 3.1. Blackleg-Phenotype Assays

**Figure 1:**
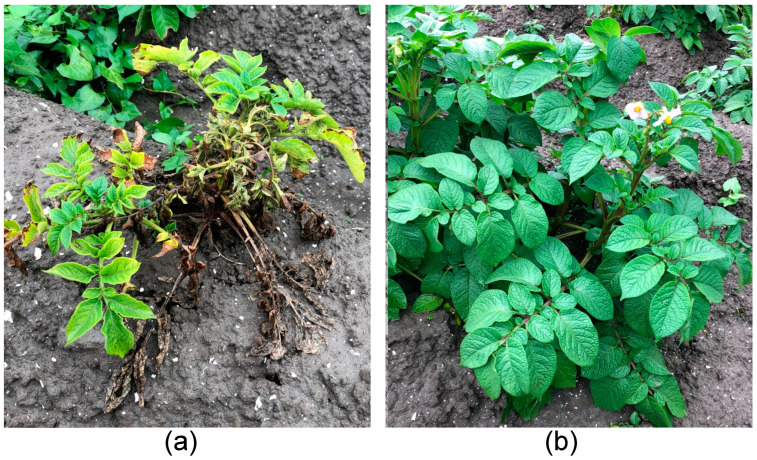
Phenotypic scoring of potato after infection with *P. brasiliense* strains. (**a**) The typical symptoms caused by a BL-causing strain. (**b**) The symptom-free phenotype.

Field experiments were conducted in three consecutive years (2018, 2019, 2020). Potato plants were assessed for disease symptoms as shown in Figure 1. Plants that showed the typical blackleg symptoms were recorded, and the percentage of diseased plants was determined for each strain. As positive and negative controls in each year, reference BL-causing and non-causative strains as well as mock-inoculated plots were included, which demonstrated that symptom expression varied slightly between years. Particularly for 2018, some background levels were observed that were adjusted for by comparing symptom expression with the mock-inoculated plants. BL-symptom scoring is included in Table S1. The blackleg-phenotype assays from 2019 and 2020 are combined with TaqMan PCR results in Section 3.3.

### 3.2. Pangenome Analysis

**Figure 2:**
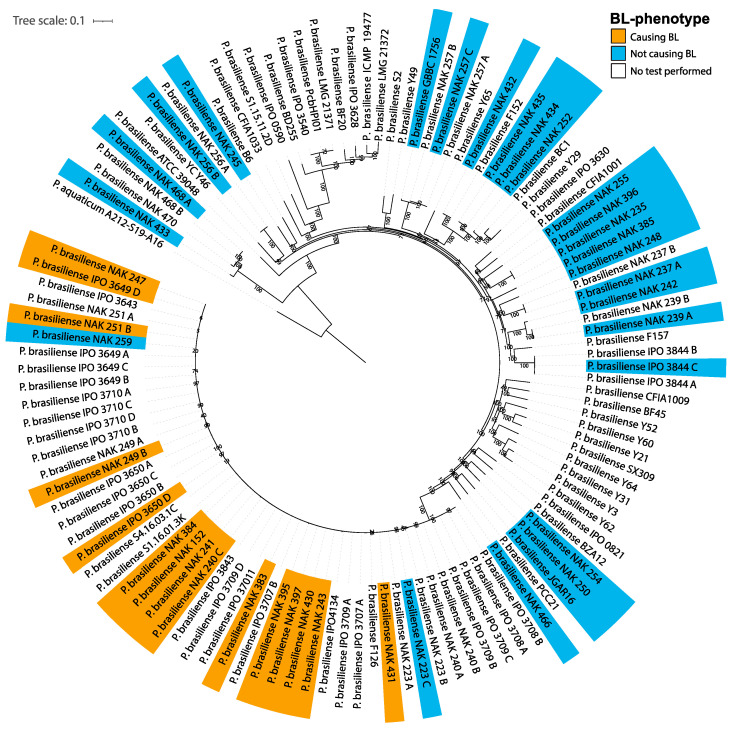
Maximum likelihood core SNP phylogenetic tree. The tree was inferred on a total of 265,343 SNPs extracted from the alignment of 2371 single-copy orthologous genes. The tree was rooted using *P. aquaticum* (A212-S19-A16) as an outgroup. Values on the branches are bootstrap support values obtained from 10,000 bootstrap replications. Strains with multiple assemblies are marked by the additional letter at the end of the tree label.

Within the pangenome comprising 117 genomes, the analysis was targeted at 40 *P. brasiliense* strains that were phenotypically characterized for BL phenotype (15 able and 25 unable to cause BL) (Table S2). In a phylogenetic representation, built from 265,343 SNPs extracted from the alignments of 2371 single-copy orthologous genes in the pangenome, the BL-causing strains are clearly grouped in a single BL-causing *P. brasiliense* clade (Figure 2). Nevertheless, strains NAK 223 and NAK 259, which were classified as non-causal in multiple years, also cluster within the BL-causing *P. brasiliense* clade (Figure 2) which thwarted the discovery of clade-specific genes associated with the ability to cause BL. When these two strains were ignored, 86 homology groups were identified; 55 of these had at least one functional annotation associated with them (Table S3). Two genes were duplicated in some strains, resulting in 88 genes. Based on the associated functions, several genes with a putative role in causing blackleg were found among proteins that were predicted to be secreted and have a possible role in pathogenesis, based on literature searches. Finally, a putative lysozyme inhibitor (LZI) and a putative Toll/interleukin-1 receptor (TIR) domain-containing protein were selected (described in more detail in the discussion).

### 3.3. TaqMan Results

**Figure 3:**
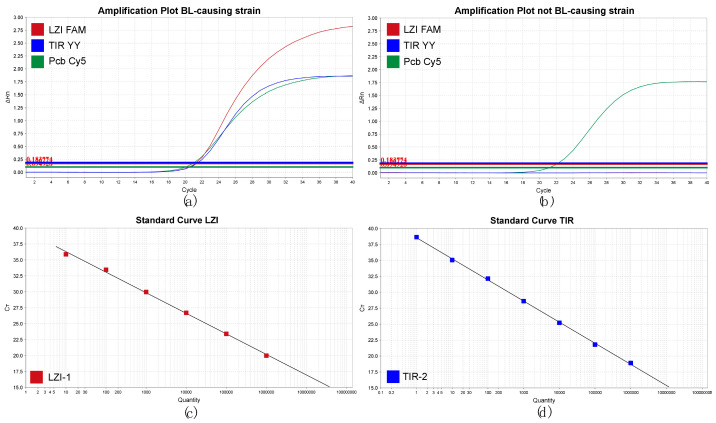
TaqMan assay for *P. brasiliense*. Upper section: classification of *P. brasiliense* strains causing or not causing BL with (**a**) BL-causing strain IPO3843 and (**b**) non-causal strain IPO3844. For each of the three targets of the triplex Pcb (green), LZI (red), and TIR (blue), the signal (delta Rn) (*y*-axis) is plotted for the PCR cycle number (*x*-axis). Lower section: standard curves showing the relationship between Ct (cycle threshold) and quantity of target DNA for LZI and TIR. (**c**) Ct values for LZI plotted against the quantity 10^1^–10^6^ in a log base 10 of the target DNA (gBlock). The slope was −3.22 indicating a PCR efficiency of 104.3%, the intercept was 39.52, and the r^2^ of the trendline was 0.998. (**d**) Ct values for TIR plotted against the quantity 10^0^–10^6^ in a log base 10 of target DNA (gBlock). The slope was −3.31 indicating a PCR efficiency of 100.6%, the intercept was 38.54, and the r^2^ of the trendline was 0.999.

The TaqMan profiles for a BL-causing strain and a non-causal strain are shown in Figure 3. Results on the purified strains and asymptomatic stolon ends are summarized in Table 2 and Table 3, respectively. Details on the Ct values of the strains are provided in Table S1. All BL-causing *P. brasiliense* strains included in the pangenome analysis were detected by both TaqMan assays. TaqMan reactions of the non-causative strains were negative with the exception of six strains. Two of the strains (NAK223, NAK259) were sequenced and included in the pangenome analysis. These were shown to be highly similar to the BL-causing *P. brasiliense* clade-based genome on the sequencing as mentioned above. TaqMan reactions of BL-causing strains were mostly positive with the exception of six strains that are genetically highly similar based on their repetitive extragenic palindromic sequence-based PCR (rep-PCR) profile. One of the isolates with the same REP profile was sequenced and showed a distinct phylogenetic position. In general, the Ct values of the TIR and the LZI assays were similar to each other and also similar to the previously described TaqMan, based on the rRNA developed previously [11] as illustrated in Figure 3a. The non-*P. brasiliense* strains were negative for the TIR and the LZI assay except for a strain identified as *P. punjabense* based on the *gapA* barcoding region that was positive for TIR and negative for the LZI and Pb assay. None of the tested *Dickeya* species were found positive for the LZI or TIR assay. Detailed results are shown in Table S1, and results are summarized in Table 2. As shown in Table 3, of 92 selected non-symptomatic potato tuber stolon-end samples, 53% of the four subsamples of fifty tubers were negative for the LZI, TIR target, and Pb. Around 60% of the subsamples that were positive for Pb were also positive for TIR and LZI. Ct values of the positive non-symptomatic subsamples range from 15.5 to 30 (=cut-off). Finally, one sample showed the Pb−, LZI−, and TIR+ combination for all four tested subsamples.

In order to determine the detection ranges of the LZI and TIR assays, 10-fold dilution series (1 to 10^6^ copies of synthetic target (gBlocks)) were prepared. In both cases, the response was linear with a slope of −3.22 and an R^2^ of 0.998 for the LZI assay, and a slope of −3.31 and an R^2^ of 0.999 for the TIR assay (Figure 3c,d).

**Figure 4:**
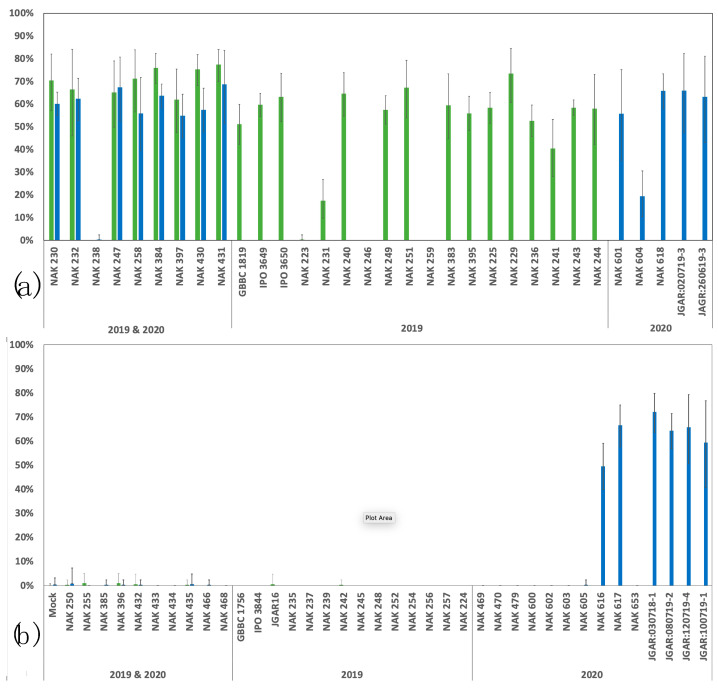
Phenotypic scoring of potato. For the specific *P. brasiliense* strains (*x*-axis) the percentage of potato plants with disease symptoms (*y*-axis) are shown. Bars indicate the observed standard deviation. Color of the bars indicate year in which the strains were tested (green 2019; blue 2020). (**a**) Strains that tested PCR positive for LZI and TIR. (**b**) Strains that tested PCR negative for LZI and TIR.

Finally, the results of the TaqMan assays on the individual strains and field phenotyping of the individual strains are combined in Figure 4. Individual *P. brasiliense* strains were positive for all three assays or only for the *P. brasiliense* assay. While in 2019, all strains that tested negative for the assay showed no symptoms, in 2020, six strains that tested negative for the LZI and TIR assays did show disease symptoms in the field. This shows that next to the dominant BL-causing lineage additional BL-causing isolates of *P. brasiliense* exist.

## 4. Discussion

### 4.1. Moving from (Sub)Species Detection to Functional Detection Using a Pangenome

The major innovation presented here is the development of an assay for diagnostics at the functional level rather than the species level. Two key ingredients were required to achieve this: a pangenomic analysis across many genomes and accurate phenotyping.

A comparative genomic strategy was used to select targets to specifically detect blackleg-causing *P. brasiliense* strains. Strains were isolated from different substrates originating from different geographic locations in the Netherlands. Most strains were isolated from potato tubers, as strong indications were found that in potato tubers both strains able and unable to cause blackleg could be present. Based on our whole genome sequence comparison, the strains comprised a wide representation of the genotypic diversity found within *P. brasiliense*. To identify links between genotype and phenotype, reliable phenotyping is imperative. Multi-year phenotyping showed consistent scoring for the ability to cause BL of *P. brasiliense* strains and clearly distinguished two classes, BL-causing and not BL-causing, albeit with some differences in disease expression levels between strains in the group of BL-causing strains. The phenotyping, as performed in the field assays, was extremely laborious but reflects the field situation more accurately than alternative in vitro or sliced tuber assays.

### 4.2. Diligent Gene Selection Based on Both Phylogeny and Function

Initially, the phylogenic studies showed that all BL-causing strains were found in only one of the at least five phylogenetic clades identified within *P. brasiliense* [12]. This is consistent with the inability to cause BL being more basal and suggested a recent adaptation or selection of BL-causing strains. Gene acquisition and loss are the main drivers in genetic diversity in *Pectobacteria* [12] and could also account for the change in the ability to cause BL. Therefore genes were searched that were unique to the clade of BL-causing *P. brasiliense* strains and which were predicted to have an effect on causing BL based on the putative function of the gene. In addition, it was assessed if such gene function could also explain (i) the quantitative differences observed among potato cultivars, (ii) the difference observed between assays on tuber slices and whole potatoes, and (iii) why non-BL-causing strains could be associated with infected potato material.

The first target that was selected based on these criteria was LZI, a lysozyme inhibitor. Lysozymes are a natural defense mechanism against bacterial pathogens and are also found in potato [20]. In addition, the heterologous expression of lysozyme was reported to reduce infection by *Pectobacteria* [21]. Finally, lysozyme inhibitors were shown to be involved in virulence [22] although information on plant-phytobacterial systems is still limited. As the lysozyme inhibitor would counteract a plant defense response, this could also account for the differences found between plant genotypes and tissues as the production of lysozymes could show variability in relation to genotypes or physiological differences in expression levels. As the lysozyme inhibitor is predicted to be secreted, also other bacteria, including *P. brasiliense* strains lacking this gene, could benefit from the production of this protein by one of the strains in the pathobiome. These would then “hitchhike” along with the BL-causing strains, which could potentially explain the frequent co-occurrence of *P. brasiliense* strains able and unable to cause blackleg.

The second target that was selected was TIR, a Toll-like receptor domain-containing protein. When Toll-like receptors (TLRs) are activated, their cytoplasmic TIR domains undergo homo- or heterodimerization. This leads eventually to the activation of antibacterial defense responses. Recent studies have described the TIR domain-containing protein from *Brucella melitensis*, *TcpB* (*BtpA/Btp1*), and shown that the protein is involved in virulence and suppression of host innate immune responses [23]. Information on plant-phytobacterial systems is lacking. In our functional annotation, several Toll-like receptor domains in *Pectobacteria* were identified, which could indicate that similar suppression of the host innate immune system exists in this genus. Also, in this case, *P. brasiliense* strains lacking this TIR gene could benefit, taking advantage of the reduced host response after infection with the BL-causing strains.

### 4.3. Robust Detection of BL-Causing P. brasiliense

The TaqMan assays for LZI and TIR were integrated with a previously developed *P. brasiliense* assay based on the rDNA sequence [11] into a triplex and could be scored unambiguously in all pure strains tested. In *P. brasiliense* strains, results with LZI and TIR assays were always in accordance, resulting in highly similar Ct values for both assays. In other *Pectobacterium* species and in field samples, occasionally discrepancies were observed between the results of the LZI and TIR assay, in which case the samples were found to be positive for the TIR assay and negative for the LZI assay. In mixed samples, such as the potato stolon ends samples, results for the LZI and TIR assays were mostly similar. In potato stolon-end samples, the ratios between the assays can be used to quantitatively dissect the components. In individual strains, the BL-causing *P. brasiliense* clade should be positive for both the LZI and TIR assay.

The BL-causing assay had a sensitivity of 0.83 and a selectivity of 0.88. This is considerably higher than the Pb TaqMan currently in use (0.5) for BL-causing *P. brasiliense,* but not yet sufficient for large-scale detection purposes. Six strains were clearly positive for the LZI and TIR TaqMan assay but did not express symptoms in the field tests. For two of these six strains, the genome was sequenced, and the pangenome demonstrated that these clustered within the vPbr clade. This discord between the positive signal in the LZI and TIR assays and the non-BL phenotype is unlikely to be caused by unsuccessful infection in the phenotyping assays. Based on the clear distinction that could be made between the BL-causing and not BL-causing phenotype, as well as the replication of the results over different years and locations, it is likely that this inability to cause BL is a stable character of specific strains within this BL-causing clonal lineage. This prompted us to study alternative hypotheses. Perhaps, these strains contain stable genetic or epigenetic changes resulting in a loss of their ability to cause BL. Based on sequence variation, no link between the two genome sequences with functional mutation or identity by descent was observed. This would indicate that the loss of this trait may not be a single event or remains undetected by sequencing as could be the case for epigenetic changes. Nevertheless, this class of strains that are unable to cause BL is unlikely to cause major problems in detection as these false positive strains represent a minor part of the population and probably show a reduced spread as they do not cause BL. In addition, it is also not clear if the loss of the ability to cause BL could have been triggered by the isolation procedure and/or growth on artificial media and therefore could be considered an experimental artifact.

A completely different problem for diagnostics is caused by the BL-causing strains that are missed by the LZI and TIR TaqMan assays. Preliminary data indicate that these belong to at least one different clonal lineage. Whether the molecular basis of the ability to cause BL of the two BL-causing lineages is similar is unclear. No shared properties (either missing or additional genes) were found in the genome content of representatives of the two lineages compared to the strains unable to cause BL. When considering that inability to cause BL is basal and the ability to cause BL is an acquired trait, one could assume that this would not be confined to a single event but could happen on multiple occasions and involve a different set of genes giving rise to different BL-causing lineages. In the case of multiple events involving different genes, it is not possible to generate a single assay that would detect all BL-causing strains.

### 4.4. Future Diagnostics

Despite the efficiency of the developed assays for the specific detection of BL-causing *P. brasiliense*, our results also showed that our understanding of the genotype–phenotype relationship is still not complete, so future refinements are necessary. First, more strains may be needed to study this genotype–phenotype association in detail, as the current data set is a subsection of *P. brasiliense* phylogenetic lineages that can cause blackleg under field conditions.

Additionally, more functional characterization of strains will allow a more comprehensive and useful detection of plant pathogens. Functional markers could be used to further understand the ability to cause BL at a molecular level. A distant homolog of the TIR gene was found in *P. peruviense*, and also in field samples occasionally TIR was found to be present in samples negative for the TaqMan assays for LZI and *P. brasiliense*. Perhaps homologs of this gene may contribute to symptom development in other *Pectobacteria*. This also shows that *Pectobacteria* are highly dynamic, and gene acquisition and loss occur frequently, as was reported before [12]. Differences between strains able and unable to cause BL may emerge at all levels of analysis. Phylogeny does not always reflect functional diversity and, to provide further insight into functional diversity, Genome Properties (GP) in combination with machine-learning methods as recently studied for the genus Pseudomonas [24] might not only provide a better classification of unknown strains, but could also identify traits that are predictive for (in)ability to cause symptoms, providing new leads for functional analysis.

Until the mechanisms involved in causing blackleg are understood and the ability to cause BL can be predicted based on the genome content, an assessment based on gene function is not yet feasible. An alternative option would be to include markers for all detected BL-causing lineages. As the acquisition is still considered a rare event, this would allow accurate detection of BL-causing strains. Nevertheless, deployment of this strategy approach requires monitoring of *P. brasiliense* populations to make sure that new BL-causing lineages are detected timely and integrated in updating the assay design.

Lastly, the high fraction of *P. brasiliense* strains unable to cause BL in the population indicates that the ability to cause blackleg is not essential to be successful. There are several explanations for this. Firstly, *P. brasiliense* can also cause soft rot, which was not assessed in this study but could represent an important ecological niche. Alternatively, as infected material usually contains different strains and species, these could play different roles in the ecological niche of the infection site constituting the pathobiome. The joined presence with *P. brasiliense* strains able and unable to cause BL as identified in this study may be significant. Also, the presence of other bacteria could be of interest. Metagenomic analysis of infected material could perhaps help to uncover such interactions. Previously, it was shown that the infection with the blackleg pathogen *P. atroseptica* caused alterations in plant physiology leading to qualitative and quantitative changes of root exudation patterns. These changes were probably responsible for the slightly altered microbial communities [25]. More research is needed to resolve the relative importance of these aspects and the dynamics and epidemiology of the BL-causing *P. brasiliense* in potato. The described assays in this study could help to dissect the different components and quantify their presence.

## 5. Conclusions

For robust detection, the use of a multilayered detection strategy is currently recommended. It consists of an assay based on the phylogenetic detection of the species or subspecies (in this case based on the 16S ribosomal sequence for *P. brasiliense* [11]), in combination with two regions for the association trait, such as the ability to cause blackleg on potato, signified by the TIR and LZI assay. The triplex described allows the efficient and quantitative screening of the targets. In the future, a design should integrate the additional BL-causing *P. brasiliense* lineages, which would further enhance the sensitivity and selectivity.

## Figures and Tables

**Table 1:** Primers, probes, and gBlocks used in this study.

Target	Name	Oligo	Sequences (5′–3′)	Reporter Dye	Quencher	Size (bp)
LZI	LZI_F1	Forw	CGGTAAGTTATGCCGCATCT			
	LZI_R1	Rev	CACTGATCTCTTTCATTTAGCCATATC			
	LZI_P1	Probe	TGGCATTACAGAATTCATTGCCAAC	FAM	ZEN/IBFQ	
	gBlock-LZI	gBlock	ATGAAGAATATCATAAGTAAAAAAACGTTTATATTTTTATC CTTAATGGCATGTAGTACGGTAAGTTATGCCGCATCTTTTG ATTGTGAAAATGGAAATTCTAAGATTGAAAAAATGATTTGT TCAAATTATACTTTGAATAGACTAGATGATTTTCTCTCTGA AAACTATAAGTTGGCAATGAATTCTGTAATGCCAAATGAAG AAAAAAGTGAAATAAAAAATTCTCAGAAGATATGGCTAAAT GAAAGAGATCAGTGTAAAGATGTTAAATGCATTGGGGTATG TATTCGAGGCGTATAG			303
TIR	TIR-F2	Forw	AGATAAACAAGCGAGGGTTGA			
	TIR-R2	Rev	ATCTATCTCCCATTTCACCCAAG			
	TIR-P2	Probe	AAATACAGCCTCCATTAGAGTTTCCC	FAM or Yakima Yellow	ZEN/IBFQ	
	gBlock-TIR	gBlock	CAAGCGATTTTCTTGTTGAGTTAGAATCAAGAATATCTGAC TTAGGATATTTATACATTGACCTTTTGCATAATAACTCTGA AGATAAACAAGCGAGGGTTGAAAATGAACTTCAACAAGCAG ATATTTTTCTTCTATTAAATACAGCCTCCATTAGAGTTTCC CCTTGGGTGAAATGGGAGATAGATACAGCAAAATCGAATAA CATCTACAATATTAAAATCAACGTAAGTCCCTCAAACATTA ATACTGTCTTTAATGAAATTCGCTTGGCTATTAC			280
Pcb	Pcb1F ^a^	Forw	CCTTACCAAGAAGATGTGTGTTGC			
	Pcb2R ^a^	Rev	CATAAACCCGGCACGCT			
	Pcb2Prb ^a^	Probe	CAAGCGCACCTGTTGATGTCATGAGTG	FAM or Cy5	ZEN/IBFQ or TAO/IBRQ	
	gBlock-Pcb_SA-NAK	gBlock	TAGAGGCCTTACCAAGAAGATGTGTGTTGCGTGAAGTGCTC ACACAGATTGTCTGATGAAAATACTGAGCAAGCGCACCTGT TGATGTCATGAGTGTAGACTCATGCTGACGCGAGCGTGCCG GGTTTATGACCTGGTGCGGAT			144

^a^ Muzhinji et al., 2020 [11].

**Table 2:** Results of TaqMan assays on purified *P. brasiliense* strains.

Strains		Total	Positive LZI/TIR	Negative LZI/TIR
*P. brasiliense*				
	Causing BL	34	30/30	4/4
	Not causing BL	34	6/6	28/28
Sensitivity			0.83	
Specificity				0.88

**Table 3:** Results of TaqMan assays for asymptomatic stolon ends: 92 selected potato samples tested in four subsamples of 50 potato heels each. The percentage for both the aggregated four subsamples (sample) and the individual subsample are shown spit in the different categories.

TaqMan Profile		Sample	Subsample
Pb−		30.4%	53.0%
	LZI+, TIR+	0.0% ^1^	0.0% ^1^
	LZI−, TIR−	100.0% ^1^	100.0% ^1^
Pb+		69.6%	47.0%
	LZI+, TIR+	73.4% ^2^	59.5% ^2^
	LZI−, TIR−	26.6% ^2^	40.5% ^2^

^1^ Percentage of the Pb negative samples. One sample (4 reactions) showed the Pb−, LZI−, and TIR+ combination. ^2^ Percentage of the Pb positive samples.

## Data Availability

The pangenome data presented in this study are openly available in 4TU.ResearchData at https://doi.org/10.4121/fb37e29c-b94b-4888-b3c8-db889bf211e2.

## Supplementary Materials

The following supporting information can be downloaded at: https://www.mdpi.com/article/10.3390/microorganisms11082080/s1, Supplementary Tables.xlsx containing Table S1: BL-phenotype scores and TaqMan assay Ct values of *P. brasiliense* strains; Table S2: Genome assemblies and their metadata in the pangenome; Table S3: Genes associated to causing blackleg in *P. brasiliense* NAK 240 (NCBI ID CP059957).
